# Computational Load Analysis of a Galileo OSNMA-Ready Receiver for ARM-Based Embedded Platforms †

**DOI:** 10.3390/s21020467

**Published:** 2021-01-11

**Authors:** Micaela Troglia Gamba, Mario Nicola, Beatrice Motella

**Affiliations:** LINKS Foundation, 10138 Turin, Italy; mario.nicola@linksfoundation.com (M.N.); beatrice.motella@linksfoundation.com (B.M.)

**Keywords:** Galileo OSNMA, software receiver, embedded platform, ARM, spoofing, authentication, Galileo open service

## Abstract

Many GNSS applications have been experiencing some constantly growing needs in terms of security and reliability. To address some of them, both GPS and Galileo are proposing evolutions of their legacy civil signals, embedding features of authentication. This paper focuses on the Galileo Open Signal Navigation Message Authentication (OSNMA) and describes its implementation within a real-time software receiver for ARM-based embedded platforms. The innovative contributions of the paper include the software profiling analysis for the OSNMA add on, along with the comparison among performances obtained with different platforms. In addition, specific evaluations on the computational load of the whole receiver complete the analysis. The receiver used for the implementation belongs to the NGene receivers family—real-time fully-software GPS and Galileo receivers, tailored for different platforms and sharing the same core processing. In detail, the paper deals with the introduction of the OSNMA support inside the eNGene, the version of the receiver executable by ARM-based embedded platforms.

## 1. Introduction

As widely demonstrated, Global Navigation Satellite System (GNSS) signals are relatively susceptible to interference [1,2,3,4], whether from natural sources, reflections from obstacles, or attacks of intentional nature. It is not surprising, in fact, that many applications have strict requirements in terms of resilient navigation. Such a need is particularly strong for all those applications defined as critical [5], either safety-critical or liability-critical, in which the user position or velocity information might be used to take actions, relevant for people safety or for legal and economic decisions [6,7].

Within this context, the research community has been spending significant effort in studying the possible consequences of structured interference, such as spoofing, and to develop efficient methods to protect against those kinds of attack [2,8,9,10,11]. A first macro-classification of spoofing countermeasures divides them in cryptographic and non-cryptographic defenses [1]. Cryptographic defenses are based on specific features added to the GNSS signals themselves [6,12,13], and non-cryptographic methods include traditional common anti-spoofing techniques suitable to standalone receivers [1,2]. In this sense, cryptographic defenses can be seen as the contributions of the system against spoofing attacks. This kind of protection, definitely mandatory for military or governmental authorized users, can be also extended to open civil signals.

As examples, both GPS and Galileo are proposing evolutions of their legacy signals, embedding features of authentication, intended as the ability of the system to guarantee the user that non-counterfeit navigation data from one of the constellation satellites is used [6].

In particular, GPS is disclosing the Chips-Message Robust Authentication (CHIMERA) solution, suitable for the GPS L1C signal [14]. On the other hand, in 2017 the Galileo program officially announced its intention to offer a free Open Signal Navigation Message Authentication (OSNMA) service [15], designed to be disseminated on the E1 Galileo band. According to [15], the OSNMA signal-in-space transmission is expected to begin in 2020. The new signal will be fully backward compatible: in fact, the performance for users who are not able or not interested in processing the authentication bits will not be affected. On the contrary, users who want to take advantage of the authentication service must update the current Galileo receivers to process the OSNMA bits and verify the authenticity of the transmitted source.

Focusing on the OSNMA, this paper describes the implementation of the Galileo authentication scheme in a real-time software receiver for ARM-based embedded platforms, including the comparison among the performance of different platforms. This paper is an extension of the article titled “Galileo OSNMA: an implementation for ARM-based embedded platforms,” presented by the same authors at the 2020 International Conference on Localization and GNSS (ICL-GNSS) [16], thereby providing a wider analysis on the compared platforms, along with a dedicated study on the complete OSNMA-ready receiver.

Both papers are based in turn on [17], which described the implementation of the software routines able to make a real-time GPS/Galileo software receiver ready for the reception and elaboration of the OSNMA service. In fact, although OSNMA-ready commercial receivers start appearing on the marketplace, e.g., [18], an independent fully-software OSNMA solution allows one to add new features and strategies, as every part of the algorithm can be easily accessed and modified, thereby speeding up the prototyping process. Furthermore, the results achieved with a research tool, such as the one proposed in this paper, provide useful guidelines that might be exploited also for the development of commercial receivers.

The receiver used for the implementation in [17] belongs to the NGene receivers family [19], a set of software tools tailored for different platforms and sharing the same core processing. Such tools have been specifically developed for research purposes. While [17] focused on the version of NGene executable by standard PCs running a Linux operating system, both [16] and this paper deal with the introduction of the OSNMA scheme inside eNGene [20], the version of the receiver executable by ARM-based embedded platforms.

More in detail, with respect to [16], two new contributions justify this extended version: First of all, two further ARM-based platforms have been included in the platforms comparison, thereby allowing for a more widely assessment of the performance. Secondly, a specific analysis on the computational load of the complete OSNMA-ready receiver is discussed hereafter.

More in detail, after this introduction, Section 2 recalls the main characteristics of the OSNMA authentication scheme, while Section 3 describes the eNGene software receiver, along with the update to the OSNMA-ready version. The results of the software profiling analysis of the OSNMA functions are summarized in Section 4, focusing on the comparison among platforms and considering different configurations of the OSNMA parameters. Such analysis is then extended to the main functions of the complete OSNMA-ready receiver in Section 5, where the processor’s computational load is also evaluated. Section 6 draws the conclusions, sketching some ideas for future activities.

## 2. The OSNMA in Brief

To ease the reading of the paper, this section briefly recalls the main concepts behind the OSNMA scheme; refer to [21,22,23,24] for more complete and detailed descriptions.

**Figure 1:**
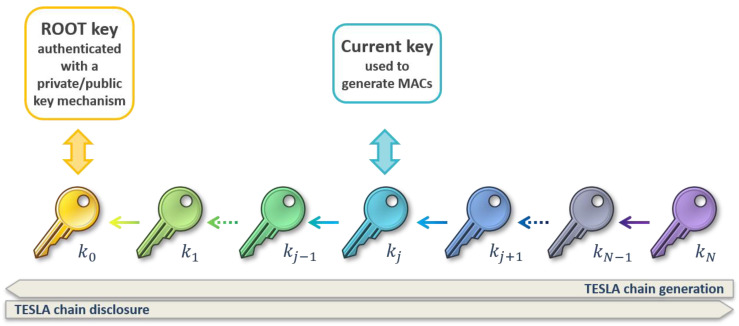
Schematic of the TESLA key chain and its use in the OSNMA scheme.

The OSNMA, as sketched in Figure 1, is based on the Timed Efficient Stream Loss-tolerant Authentication (TESLA) protocol [25], which is structured into two elements: (i) the transmission of a message authentication code (MAC), used to authenticate the plaintext navigation message, and (ii) the delayed transmission of the key used to compute the MAC.

The delayed release mechanism ensures that the key is not known until after the message and the MAC are received. The protocol also prevents the spoofer from generating messages, keys and MACs, and broadcasting them compliant to the specifications. For this, the key belongs to a chain of keys, referred to as TESLA key chain, in which each key is generated from the previous one with a one-way function. The generation of a chain of length N starts with a random secret key $k_{N}$, and ends with a public root key $k_{0}$, certified as authentic. The disclosure of the chain occurs in the opposite order. The root key $k_{0}$ is transmitted along with a digital signature generated using a standard asymmetric scheme, based on a pair of private and public keys: the receiver can use the digital signature and the public key to check the authenticity of the received $k_{0}$.

The receiver, once certified the root key as authentic, can start the authentication verification process, which is basically performed by two steps:

1. The current received TESLA key is authenticated with the root key, by performing the one-way function the required number of times. Alternatively, if one or more authentication verifications have been already successfully occurred, the current key can also be authenticated with a previous key from the chain, closer than the root key.
2. The MAC is then regenerated using the current key and the navigation data. If it coincides with the previously received MAC, the navigation data are authenticated.

The OSNMA information will be transmitted in the E1B Galileo I/NAV message [26], using the 40 bits marked as “Reserved 1” in the odd page part, thereby providing an equivalent bandwidth of 20 bps, for a total of 600 bits every I/NAV subframe.

The authentication information is sent in two sections transmitted in parallel:

- The HKROOT section that contains the global headers and the digital signature message (DSM), usually signing a root key (8 bits per page);
- The MACK section containing the MACs and the associated delayed keys (32 bits per page).

The OSNMA will be transmitted only from a subset of satellites, able to cross-authenticate also those satellites whose signals do not carry the OSNMA information.

## 3. Development Work

The NGene receivers represent a good set of choices for the implementation and testing of the OSNMA functionality on different platforms, including standard PCs [17,19], ARM-based embedded platforms [20], and Android smartphones [27]. Apart from the adaptations needed by the different platforms, all the receivers belonging to the NGene portfolio share the same core processing and features:

- The real-time capability of processing the GPS, Galileo and EGNOS signals broadcasted on the L1/E1 band;
- The implementation of the whole GNSS signal elaboration chain, from the acquisition to the position velocity and time (PVT) computation;
- The Software-Defined-Radio (SDR) approach, which offers the highest level of maintainability, flexibility and portability of a fully software implementation;
- The support for several L1/E1 radio frequency (RF) USB front ends (FEs) [28,29,30,31], allowing the user to also specify a custom FE.

In particular, most of the functionalities are coded in ANSI-C, allowing for a high level of portability among different operating systems (OSs) and platforms, and only the modules that have to process high data rates, such as the samples coming from the FE at tens of megahertz (e.g., 1–16 MHz), are coded in assembly language, exploiting processor-specific optimizations.

**Figure 2:**
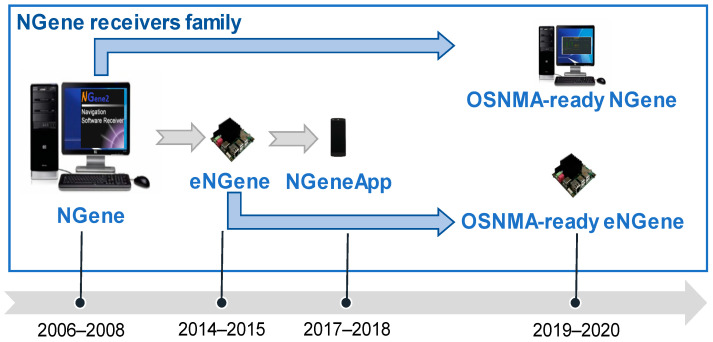
NGene: the set of receivers belonging to the family and their derivation.

Both the receivers considered in this paper, i.e., NGene [17] and eNGene [20], require a Linux operating system, but while the former can be executed by standard PCs, the latter is tailored to ARM-based embedded platforms, as better described in the next subsection. Figure 2 shows an illustrative picture of all NGene family’s receivers developed along more than one decade.

### 3.1. eNGene

eNGene has been obtained porting the original code of NGene to an ARM-based embedded platform. During the porting operation, special care has been devoted to the translation of the functionalities coded in assembly language from the Intel processor’s instruction set to the ARM one. In addition to this translation, one of the main noteworthy differences of eNGene with respect to NGene is the multiple-threads architecture. Indeed, eNGene explicitly splits functions into different threads, thereby fully exploiting all the cores available in the ARM processor, optimizing the load to enhance the real-time capability. The eNGene architecture is compatible with almost every ARM-based embedded platform, without the need for any adaptations, since it does not exploit any specific characteristic of the embedded board (e.g., FPGA based hardware accelerators).

### 3.2. The OSNMA Add-On

The OSNMA functions, already implemented in NGene, as reported in [17], have been included also in eNGene. Being low data rate functions that elaborate the navigation message decoding output, they have been coded in ANSI-C and exploit the C-based open-source OpenSSL 1.1.1 library [32] for the cryptographic operations, thereby considerably easing the porting procedure. The OpenSSL 1.1.1 library is available with both the binary files and the source code, easing the installation on different operating systems and platforms.

**Figure 3:**
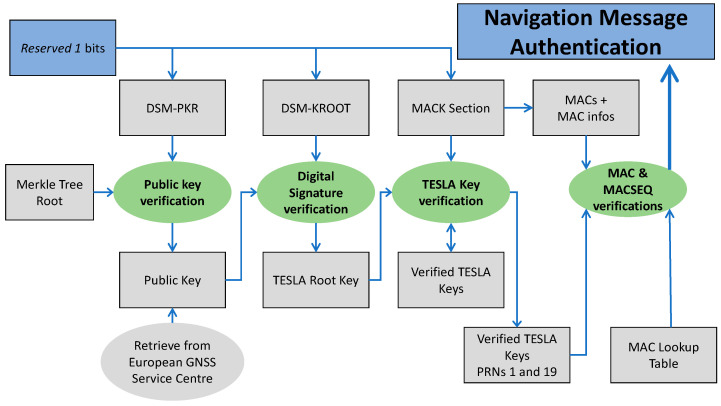
Simplified block scheme of the main OSNMA functionalities implemented in the receiver, adapted and restyled from [17].

The main OSNMA functionalities implemented in the receiver are depicted in Figure 3, while a short description is reported in Table 1, together with the list of cryptographic functions, main required inputs and outputs produced by each function.

### 3.3. The Platforms Used for the Performance Analysis

In this paper, the profiling analysis of the OSNMA ready receiver is described for three ARM-based boards, and for reference, a standard Intel-based desktop PC.

The platform originally used for the implementation of eNGene was an ODROID-X2 [33], which entered the market in 2012 and was discontinued in 2015. The ODROID-X2 was a powerful, low cost and pocket-sized board, featuring a 1.7 GHz Quad Core ARM Cortex-A9, 2 GB RAM memory and a number of peripherals, such as a high-definition multimedia interface (HDMI) monitor connector and six USB ports, which can be used for keyboard, mouse and FE. The board hosts an Ubuntu Linaro OS distribution, booting from an embedded Multi Media Card (eMMC), so that the developer can work directly on the target platform using Eclipse IDE and GNU Compiler Collection (GCC) compiler.

The performance obtained with ODROID-X2 has been compared with those gotten from two more recent boards, namely, the Raspberry Pi 4 [34] and ODROID-C4 [35]: while their general architecture is similar to that of ODROID-X2, they exhibit newer ARM processors: indeed, Raspberry Pi 4 uses an ARM Cortex A72 and ODROID-C4 uses an ARM-Cortex A55. Both processors implement the ARMv8 64-bit instruction set, whereas the ARM Cortex A9 on ODROID-X2 supports the ARMVv7 32-bit instruction set. The factory configuration of the Raspberry Pi 4 uses the ARM processor as a 32-bit processor, so the eNGene developed for ODROID-X2 is fully compatible with the new board. On the contrary, ODROID-C4 required a rewriting of the assembly parts to allow the execution on the 64-bit processor, due to the different assembly syntax of the 64-bit instruction set with respect to the 32-bit one.

**Figure 4:**
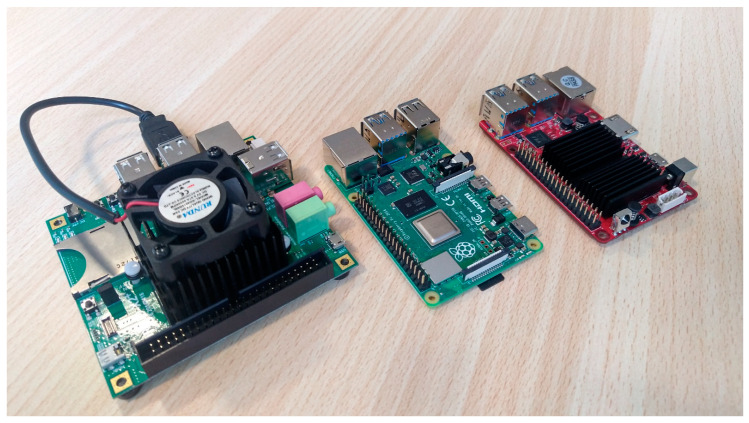
The ARM-based platforms used in the tests, from the left: ODROID-X2, Raspberry Pi 4, ODROID-C4.

Figure 4 reports a picture of the used platforms, and Table 2 summarizes their main hardware features. In particular, the first column reports the reference desktop PC characteristics, whereas the other three columns show the features of the three ARM-based platforms.

## 4. Software Profiling Analysis of the OSNMA Functions

The software profiling, already performed to evaluate the OSNMA additional computational cost for the NGene receiver in [17] and eNGene on ODROID-X2 in [16], has been extended to the two additional embedded boards, i.e., Raspberry Pi4 and ODROID-C4.

### 4.1. Simulation Set-Up

The analysis is mainly focused on the cryptographic functions call rate and execution times. The memory occupation has already been evaluated in [17], resulting in a negligible additional memory occupation due to the OSNMA functionality, with respect to the classical data processing.

The same testbed as in [16,17] has been used: it only includes the data decoding and the OSNMA functions, and all the related data structures, thereby speeding up the profiling analysis. The Galileo OS navigation message including the OSNMA bits has been generated by a MATLAB^®^-based script and provided as input to the testbed.

Table 3 summarizes the navigation message generation setup, where four sets of values have been considered, corresponding to all the four Elliptic Curve Digital Signature Algorithm (ECDSA) EC options, as indicated for the NPKT field. The digital signature verification is indeed the heaviest function from a computational point of view [17]. The four different configurations imply that the reception of complete DSM-KROOT and the reception of DSM-PKR requires different numbers of subframes, as specified by NB_KROOT and NB_PKR fields. All other parameters have been kept fixed, including the number of MACK blocks per subframe (see NMACK field), and key (KS field) and MAC (MS field) sizes. In the chosen configuration, OSNMA transmits two MACK blocks per subframe, including five 10-bit MAC fields and one 96-bit key per block. Finally, a short TESLA chain length has been considered, as indicated by the D_KROOT parameter.

The software testbed has been executed on the Raspberry Pi4 and ODROID-C4 platforms for a total of about 6 h of running time for each set of values reported in Table 3.

### 4.2. Analysis Results

Table 4 reports the achieved profiling results in terms of call rate and execution times for all the four platforms indicated in Table 2 using the set of parameters S1 in Table 3. In particular, for each listed OSNMA function, Table 4 reports the call rate, along with the statistical analysis of the execution times on the four target platforms, i.e., the mean value avr, the standard deviation σ and the estimated accuracy ε, evaluated as the ratio between the mean value and the standard deviation. In Table 4, the OSNMA functions are presented in a decreasing order of call rate, thereby showing the MAC and TESLA key verifications at the top of the list, while the digital signature (DS) and public key verifications are at the bottom. Concerning the public key verification, it is worth noticing that, although the DSM-PKR transmission rate has not been specified yet, it is assumed to be very low in nominal conditions [25,26]. Additionally, to remove the dependence of the TESLA key verification on the number of steps to be traversed in the chain (from the key to be verified back to the last verified key), the profiling of one step of TESLA chain has been reported.

Looking at the statistical analysis of the execution times, a clear degradation can be observed for all the Cortex-A processors, due to the reduced computational performance of the embedded platforms with respect to the PC. As detailed in Table 5 the mean values of degradation range from a factor of 4.3 to 9.2 for all the functions, except the digital signature verification, which shows degradations by a factor of 10 to 21, thereby resulting in the heaviest load. Standard deviation increments are even bigger, varying from 3.5 to 29 times. Those achieved by the Cortex-A9 in particular, are inversely proportional with respect to the mean execution time, translating into a higher estimation inaccuracy for functions with a shorter execution time (see the TESLA key verification for platform 2 in Table 4).

This behavior is likely due to the ODROID-X2 Ubuntu Linaro task scheduler. Now, such a trend cannot be observed for Cortex-A72 and Cortex-A55 processors (see platforms 3 and 4 in Table 4), where short execution times do not necessarily imply higher measurement inaccuracies. This can be explained considering the different operating systems (OSs), i.e., Raspbian and Ubuntu Mate, featured respectively by Raspberry Pi4 and ODROID-C4. Furthermore, for both platforms better mean execution time performance with respect to ODROID-X2 can be appreciated, especially for high load functions. The newer platforms show similar performance for all the functions, excluding the digital signature verification, which exhibits improvements of 34% and 50% for platforms 3 and 4 respectively.

Being the heaviest function, the digital signature verification deserves some more investigations, particularly focusing on the embedded platforms, whereas the standard PC performance does not result to be critical. In this regard, Table 6 reports the detailed profiling analysis of the main subroutines, including the digital envelope (EVP) application programming interface (API), the verified context routines provided by the Open SSL library and an encoding function to make the public key compliant with the input format required by the EVP functions. In particular, verifying a message requires a three-stage process: initialize the verification context with a message digest/hash function and public key (EVP Verify Init), add and hash the message data (EVP Verify Update) and finally, verify the data against the received signature (EVP Verify Final). As can be observed in Table 6, the main contribution is given by EVP Verify Final, meaning that the whole computational complexity resides in the low-level EVP API implementation. This is true not only for the ODROID-X2 platform, as already shown in [16], but also for both the newer boards, reported for completeness. Although somehow expected, since all processors belong to same family, i.e., ARM Cortex-A, such results confirm that the lower level implementation of EVP library APIs and their behavior in reaction to calls are the same for all the considered boards. This means that the developer has no chance to further optimize this function, other than implementing it from scratch. This last approach would be time consuming and not convenient from a security point of view; the usage of a different cryptographic library will be probably more affordable.

**Figure 5:**
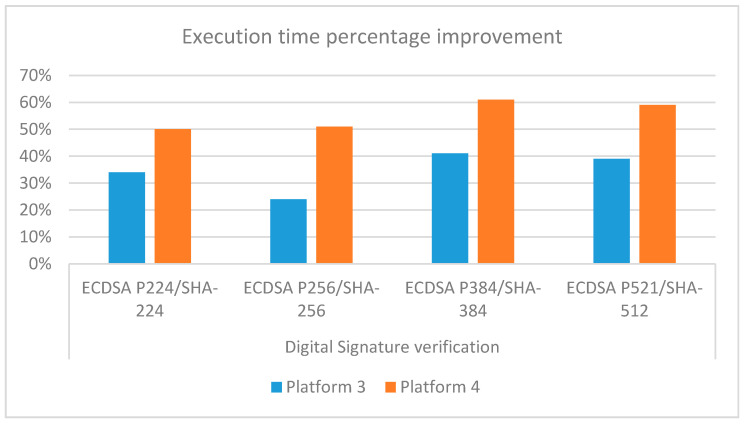
Execution time percentage improvements I for platforms 3 and 4 with respect to platform 2 for all the four ECDSA options of the digital signature verification in Table 3.

Table 7 completes the profiling analysis of the digital signature verification for all the four sets of parameters values S1 to S4 indicated in Table 3 and the newer embedded platforms. Results for the ECDSA-P224/SHA-224, already shown in Table 4 and Table 6, are also reported to ease the comparison. The mean execution time increases for higher EC orders, except for the P256, which shows the best performance. This could be due to the fact that the NIST P-256, also known as prime256v1, is the most preferred elliptic curve used nowadays on the Internet and the default one for OpenSSL, so that it was likely subject to a specific low-level optimization [36]. Figure 5 summarizes the percentage improvement I for the newer boards with respect to ODROID-X2. I is defined as $I=\frac{\left(avr_{p2}-avr_{px}\right)}{avr_{p2}}*100$, where subscripts p2 and px indicate platform 2 and platform x={3, 4} respectively. It can be noticed how ODROID-C4 exhibits better performance (I ranging from 50% to 61%) than Raspberry Pi4 (I ranging from 24% to 41%).

### 4.3. Some More Considerations about the Real-Time Compatibility

Apart from P256, results for the other elliptic curves pose some concerns about their compatibility with real-time execution. In particular, as better detailed in [17], the receiver main loop elaborates 1 ms bunches of samples for each channel, i.e., satellite, meaning that the cumulated elaboration time of the whole signal processing chain, including acquisition and tracking for all the channels, OSNMA support and other operations, cannot exceed 1 ms. This means that functions with very high computational burdens might cause the loss of input samples and compromise the real-time capability. Despite one step of TESLA chain exhibiting a very low execution time, as shown in Table 4, the total computational load required to verify a TESLA key depends on the number of steps to be traversed in the chain, as mentioned before. As soon as the first received TESLA key has been verified, a maximum of NMACK × NS steps has to be performed in each subframe. At the power on stage, instead, the receiver has to cross the chain back to the root key, implying a huge burden in case of a long TESLA chain, e.g., more than six million steps in the current configuration reported in Table 3 if the root key dates back one month. This aspect has been already fully addressed in [17], for which the implementation of a strategy based on the workload spreading over time has been carried out in order to preserve the real-time capability of the receiver. Such a strategy could be extended also to other OSNMA functions, such as the digital signature verification. In addition to this, a reduction of the call rate could also be evaluated, for instance, avoiding repeating the key root authentication unless a chain or public key renewal occurs. The same approach could be used to limit the number of MAC verifications, in the case of excessive computational burden.

## 5. Computational Burden Analysis of the Complete Receiver

In order to check the compatibility with the real-time implementation, an analysis of the computational load of the complete OSNMA-ready receiver is presented hereafter.

### 5.1. Test Setup

The tests of the complete receiver signal processing chain have been carried out by feeding the navigation message employed in the OSNMA functions profiling to a NAVX-NCS professional GNSS signal generator [37]. Table 8 summarizes the main parameters of the test setup. In particular, for the Galileo signals’ generation, the navigation message with the set of values *S4* in Table 3 was used, thereby requiring the heaviest load for the digital signature, as shown in the previous section. The generated RF signal is then given as input to a RF FE, whose configuration is reported in Table 8.

Two test campaigns have been carried out: in the former the receiver was launched in post-processing mode, thereby reading the file of raw samples previously grabbed, and in the latter it was launched in real-time, thereby elaborating on the fly the RF signal. In particular, the former configuration was needed to perform the software profiling analysis of the main receiving functions, whereas the latter one was adopted to measure the real-time processor load required by the receiver over time. The next two subsections report the results achieved during the two test campaigns.

### 5.2. Profiling Analysis of the Complete Receiver Chain

In order to perform the profiling of the complete receiving chain, the receiver has been fed by a 10 min file of raw samples, configured to elaborate 12 satellites, i.e., six GPS and six Galileo, and executed iterating six times, for a total of about 1 h of equivalent running time on all four platforms listed in Table 2.

**Figure 6:**
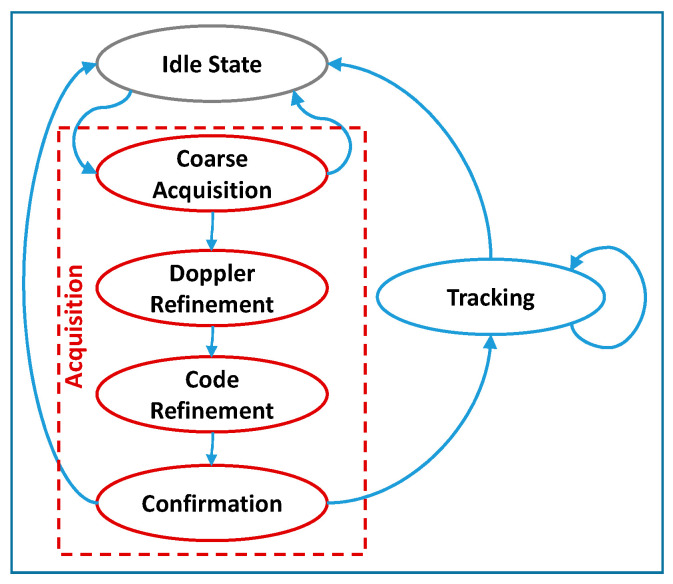
Simplified block diagram of the channel finite state machine of the NGene family receivers, adapted and restyled from [17].

Table 9 reports the average execution time required by the main processing steps performed to elaborate one code period of input GNSS samples, i.e., 1 ms for GPS and 4 ms for Galileo, and compute the PVT. In particular, as better detailed in [17], and recalled hereafter for the reader’s convenience, the receiver main loop elaborates 1 ms bunches of samples for each channel, i.e., satellite, through a finite state machine, illustrated in Figure 6. Such finite state machine includes acquisition, further detailed in coarse acquisition, Doppler and code refinements and confirmation, and tracking for all the channels. The tracking includes the calls to the Galileo OSNMA functionalities.

In a global picture, the PVT computation certainly represents the heaviest processing step, as expected; besides, its very low call rate makes it less critical than the channel state machine’s operations, showing call rates of at least three orders of magnitude higher. The coarse acquisition is the processing step that requires the highest execution time and the highest call rate, as already pointed out in [20], making it the most demanding one for a real-time execution. It is worth noticing that the impact of the OSNMA verifications on the Galileo tracking burden was expected to be negligible on average, due to their much lower call rate. This was confirmed by the achieved result, which shows it to be comparable to the Galileo tracking without OSNMA shown in [20] for the standard PC and ODROID-X2.

**Figure 7:**
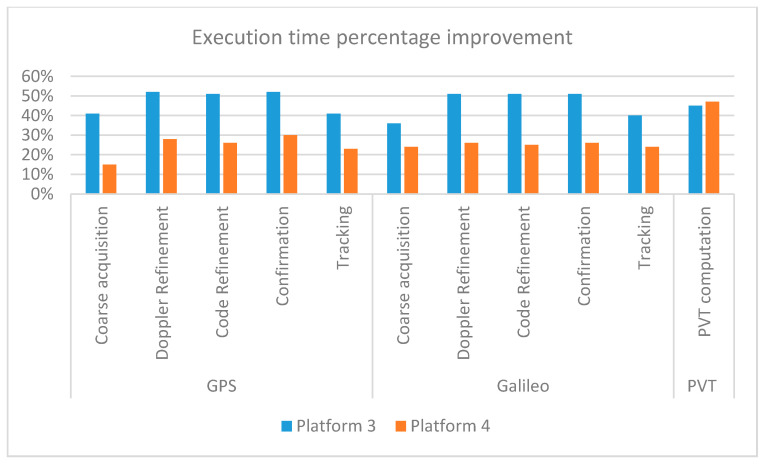
Execution time percentage improvement I for platforms 3 and 4 with respect to platform 2 for all the main processing steps reported in Table 9.

As expected and already observed in Section 4, the standard PC outperforms all other platforms: the performance degradations in terms of execution time are reported in Table 10 and roughly range in the intervals 13–16 times, 6–9 times and 8–13 times for all the processing steps in the ODROID-X2, Raspberry Pi4 and ODROID-C4 machines, respectively. Furthermore, differently from what observed in Section 4 where the newer platforms basically showed similar performances with the advantage of ODROID-C4 being limited to the digital signature verification, here Raspberry Pi4 (platform 3) overcomes ODROID-C4 (platform 4), as is clearly visible in Figure 7 with improvements from 16% to 34%, except regarding the PVT computation with a degradation of about 4%. Although the two boards, namely, platforms 3 and 4, have somewhat similar hardware features, as reported in Table 2, the different results could be partially explained by the final purposes of the two different processors: indeed, as declared by the manufacturer, Cortex-A72, released in 2015, is a high single-threaded performance CPU, whereas Cortex-A55, released in 2017, targets more high power-efficiency mid-range applications. Another aspect to be taken into account is that, as described in Section 3.3, the original 32-bit ARM v7 assembly code has been rewritten into the equivalent 64-bits ARM v8 assembly to make it compatible with ODROID-C4, mainly focusing on the direct translation of the assembly instructions, rather than on the full exploitation of the instruction set features offered by the new architecture. A specific optimization phase could likely reduce the performance gap.

### 5.3. Real-Time Processor Load Analysis

The profiling presented in the previous subsection provides an estimation of the computational weight of each software function, allowing to identify any possible bottleneck that can then be addressed by the developer to improve performance. Anyway, a software profiling alone cannot show a whole and final picture of the real time performance. Indeed, the total receiver computational load is directly dependent on the number of satellite signals to be simultaneously elaborated. Thus, an analysis of the processor load performed on the application in real-time is required to evaluate the limit each platform is able to reach.

For this test campaign, the receiver was directly fed by the output of the RF FE, thereby processing on the fly the generated raw digital samples.

**Figure 8:**
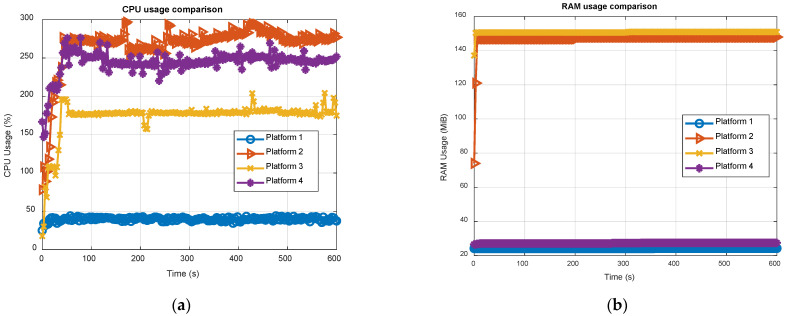
Time evolution of the CPU (**a**) and RAM (**b**) usage of eNGene receiver on the four platforms for 12 satellites, i.e., 6 GPS and 6 Galileo, simultaneously tracked.

Figure 8 reports the CPU and RAM usage of the software receiver, running in real-time on all the four platforms for 12 satellites, i.e., six GPS and six Galileo, simultaneously tracked, across 10 min of total execution time. The results were obtained using the Linux utility top. It is worth noticing that eNGene features a multi-thread structure, exploiting all available cores hosted by the embedded platforms, i.e., four, as shown in Table 2, implying a CPU usage ranging from 0% up to 400% in Figure 8. On the contrary, running on a standard PC, NGene is a single-core process, with no need to specifically split the processing on all the eight available cores.

Results in Figure 8a are in line with the profiling results described in the previous subsection. Indeed, with a usage less than 50% of the total Intel CPU power, the standard PC (see the light blue plot) exhibits performance far superior than any other platforms. Among the embedded boards, ODROID-X2 (red plot) shows the worst result, as expected, and Raspberry Pi4 (yellow plot) overtakes ODROID-C4. The RAM usage, expressed in MiB unit (1 MiB = 2^20^ bytes), deserves a separate discussion. The presence of two main clusters can be clearly noticed in Figure 8b: a former around 25 MiB for the standard PC and ODROID-C4 (both with a 64-bits OS) and a latter around 150 MiB for the remaining boards (both with a 32-bit OS). Such difference is not negligible and requires further investigations. In this regard, it is worth noticing that top does not report only the memory statically or dynamically allocated by the program, which is the same on all the considered boards, but the so-called resident memory or resident RAM, defined as the non-swapped physical memory a task is currently using, including all stack and heap memory, and memory and pages actually in memory from shared libraries. According to this, it is clear how the RAM usage strictly depends on the OS and is not in the direct control of the developer. Anyway, as a general observation, and considering the increasing size of available RAM abord the modern processors, the observed values cannot be considered critical.

Table 11 summarizes the CPU and RAM usage results, showing the average and maximum CPU load, and the maximum RAM usage. The Raspberry Pi4 is the one getting the best results among the embedded boards, showing 28% and 26% improvements with respect to ODROID-C4 in terms of average and peak CPU usages respectively, thus being perfectly in line with the results shown in Figure 7. On the other hand, ODROID-C4 exhibits a much more efficient RAM usage, but, as already said, this is not a critical indicator for the real-time execution.

**Figure 9:**
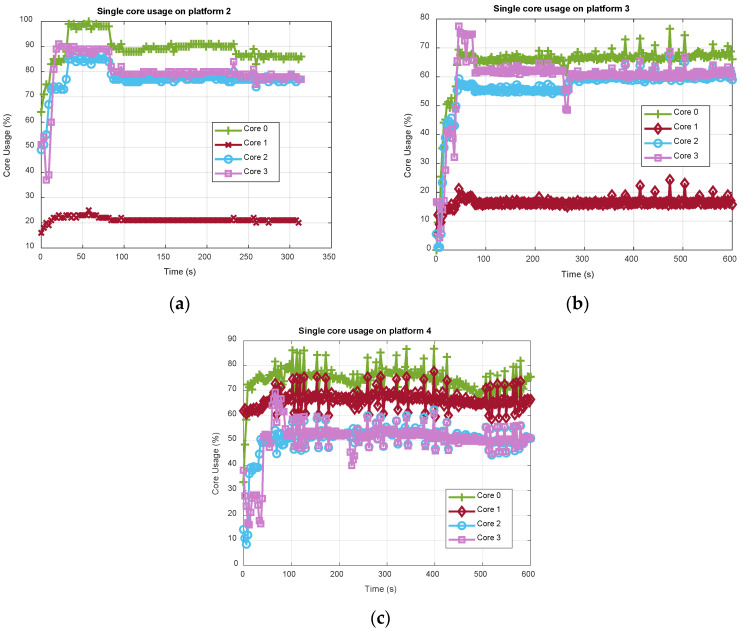
Time evolution of the cores’ usage of eNGene receiver on ODROID-X2 (**a**), Raspberry Pi4 (**b**) and ODROID-C4 (**c**) platforms for 12 satellites, i.e., 6 GPS and 6 Galileo, simultaneously tracked.

Now, focusing on the embedded boards only, Figure 9 shows how the CPU usage reported in Figure 8 distributes over each single core. In this regard, with N being the total number of configured channels (total GPS and Galileo), the GNSS processing is allocated on the four cores as follows:

- Core 0 is scheduled for the execution of the main thread, in charge of performing the main function, the tracking of $N_{0}=\left\lfloor \frac{N}{3}\right\rfloor$ number of channels and the PVT computation.
- Core 1 is devoted to the FE thread, in charge of managing the FE and USB only.
- Core 2 is allocated to a channel thread, dedicated to tracking $N_{2}=\left\lceil \frac{N}{3}\right\rceil$ number of channels.
- Core 3 is allocated to another channel thread, dedicated to acquiring 1 channel and tracking $N_{3}=N-N_{0}-N_{2}$ number of channels. It is also in charge of executing the TESLA key verification thread, created only for the first received TESLA key in case the distance in the chain from the root key is above 700 steps.

The interested reader can refer to [20] for details about the multi-thread structure of eNGene. The above-described channels-cores mapping rule is totally empirical, and for the considered test setup where N=12, cores 0, 2 and 3 elaborate $N_{0}=N_{2}=N_{3}=4$ channels each. With the acquisition being the heaviest function from a computational point of view, as already said, only one channel is acquired at a time and always allocated on a specific core, i.e., 3.

**Figure 10:**
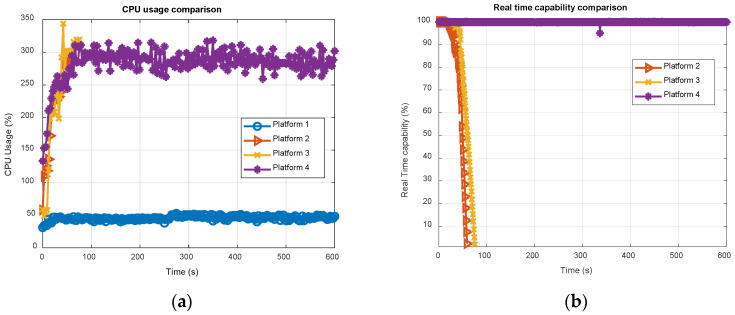
Time evolution of the CPU usage on the four platforms (**a**) and real-time capability of eNGene receiver on the three embedded platforms (**b**) for 16 satellites, i.e., 6 GPS and 6 Galileo, simultaneously tracked.

Looking at Figure 9, as expected the core 0 (green plot with “plus” markers) is the one showing the highest CPU usage, since it performs PVT computation in addition to the tracking of four channels, whereas cores 2 (light blue plot with “circle” markers) and 3 (purple plot with “square” markers) have similar loads, being dedicated to the tracking of the same number of channels. The slight increase of the core 3 load, particularly visible in the first part of the test in Figure 9a,b, is justified by the acquisition stage. Once all channels have been successfully acquired and are in the tracking loop, no additional channel is scheduled for acquisition, unless a tracking lost occurs. Finally, the core 1 load roughly stands at around 20% for both ODROID-X2 (Figure 9a) and Raspberry Pi4 (Figure 9b), whereas it is much higher, roughly around 65% for ODROID-C4 (Figure 9c). Again, a clear difference of the 32 bit and 64 bit OS behavior is evident as for the RAM usage. Now, eNGene makes use of the libusb library to manage the USB stream of raw samples from the FE; thus such behavior could be likely due to a different libusb library implementation and handling from the OS side. It is worth noticing that, although specifically and properly setting both thread-cores mapping and the threads priority with root permissions at the receiver power on, the developer has not full control of the OS task scheduler. This probably suggests implementing a real-time core load check, thereby changing dynamically the allocation of the tasks. Indeed, while the load among cores looks more balanced on ODROID-C4, it seems core 1 could bear additional tasks on ODROID-X2 and Raspberry Pi4. The requirement for a better balance can also be deduced looking at the results in Figure 10a, where the number of channels has been increased to 16, i.e., 10 GPS and 6 Galileo, while keeping the same cores allocation (five channels on cores 0 and 3, and six on core 2). It can be observed how, among the embedded boards, only ODROID-C4 is able to bear such a load, whereas both ODROI-X2 and Raspberry Pi4 stopped working before 80 s for a buffer overrun error. Indeed, as better detailed in [20], an intermediate buffer is used to store the samples coming from the USB FE and makes them available for the channels’ elaboration, in the so-called producer-consumer relationship. When new samples from the USB FE are available but the intermediate buffer does not have free locations, a buffer overrun happens. The percentage of available memory locations in the intermediate buffer is therefore a good indicator of the real-time capability of the receiver, as reported in Figure 10b for the embedded boards only.

Other aspects that should be carefully considered are the different clock values and speed controller policies (governors) of the processors under consideration. For instance, ODROID-X2 has been overclocked to 2 GHz [20] and the governor set to performance, while no specific setup has been forced on Raspberry Pi4 and ODROID-C4 (default governor set to on-demand and performance respectively).

As is clearly visible from the above analysis, the maximum number of signals simultaneously tracked (and acquired) in real-time strictly depends on the platform, since the software functions’ execution time relies on the computational power offered by the specific processor, and on the OS task scheduling policy. In this regard, some duration tests have also been performed running the receiver for days: such tests confirm that ODROID-C4 (platform 4) is able to bear up to 16 satellites: this can be considered as a maximum limit in this current setup and for this specific platform. That limit drops to 12 for both ODROID-X2 (platform 2) and Raspberry Pi-4 (platform 3). Anyway, as pointed out above, a possible performance improvement for the platforms 2 and 3 cannot be excluded when implementing, for instance, a different task-core allocation rule, currently under investigation. According to this, newer and possibly more powerful boards are expected to be able to bear at least 12 satellites, which could be considered somehow a lower bound of general validity.

## 6. Conclusions

This paper presents the software profiling analysis of the OSNMA functions implemented in a real-time GNSS software receiver targeted for ARM-based embedded platforms. This analysis has been performed for three different embedded platforms, and a standard PC has been used as a reference.

The first step of the analysis is about the computational burden of the basic OSNMA functionalities (TESLA key verification, MAC verification, MACSEQ verification, digital signature verification, public key verification). The execution times measured for the embedded platforms show a performance degradation that ranges from 4 to 21 with respect to the execution time recorded on the PC. The functionality that exhibits the worst degradation is the digital signature verification, whose complexity increases with the EC order, except for the P256 curve, which shows the best performance, likely due to a low-level implementation optimization of the used cryptographic library function.

The second step of the analysis is the assessment of the compatibility of the Galileo OSNMA implementation on ARM-embedded boards with the real time elaboration of the GNSS signal: a complete profiling has been executed, including all the steps of the GNSS signal elaboration (e.g., acquisition, tracking, PVT computation).

All the reported results demonstrate that the OSNMA support does not impair the real-time capability of the ARM-based implementation, especially when the most recent platforms are considered. At the same time, the need for an optimized scheduling of the multi-thread architecture of the receiver has been demonstrated by the real-time tests, which have been strongly affected by the different operating systems of the considered platforms.

## Figures and Tables

**Table 1:** OSNMA functions’ descriptions. For each one the list of cryptographic functions, main inputs and outputs are also shown. © [2020] IEEE. Reprinted, with permission, from [16].

Function	Description	Crypto Functions	Main Inputs	Validated Outputs
**Digital Signature verification**	verification of the root key received in the DSM-KROOT message, by means of the Elliptic Curve Digital Signature Algorithm (ECDSA) with several EC and hash functions options	ECDSA-P224/SHA-224 ECDSA-P256/SHA-256 ECDSA-P384/SHA-384 ECDSA-P521/SHA-512	DSM-KROOT message public key	TESLA root key
**TESLA key verification**	verification of a key received in a MACK block, applying a hash function for a number of times equal to the distance in the TESLA chain between the key to be verified and the last verified key or the root key	SHA-256 SHA3-224 SHA3-256	TESLA key Galileo System Time (GST) last verified TESLA key (or root key)	TESLA key
**MAC verification**	verification of the MAC/MAC0 field received in the MACK block	HMAC-SHA-256 CMAC-AES	I/NAV navigation message Galileo System Time (GST) MAC and MAC-Info fields a verified TESLA key (PRN1 or PRN19)	specific portions of the navigation data, depending on the Authentication Data and Key Delay (ADKD) field
**MACSEQ verification**	verification of the MACSEQ field in the MACK block, similar to the MAC verification		MAC lookup table Galileo System Time (GST) MACSEQ and MAC-Info fields a verified TESLA key (PRN1 or PRN19)	MAC-Info
**Public key verification**	verification of a new public key received in a DSM-PKR message, through the Merkle Tree	SHA-256	DSM-PKR message Merkle Tree Root	public key

**Table 2:** Platforms used for the software profiling analysis. © [2020] IEEE. Extended, with permission, from [16].

Platform	Platform 1 [16,17]	Platform 2 [16]	Platform 3	Platform 4
Board	Dell Precision T1700 Desktop PC	ODROID-X2	Raspberry Pi 4	ODROID-C4
Processor	Intel Xeon E3-1270 v3	Samsung Exynos4412 ARM Cortex-A9	Broadcom BCM2711 ARM Cortex-A72	Amlogic S905 × 3 ARM Cortex-A55
Base frequency of the processor	3.50 GHz	1.7 GHz	1.5 GHz	2 GHz
Cores	8	4	4	4
Memory	16 GB DDR3	2 GB DDR2	4 GB LPDDR4	4 GB DDR4
Storage	1 TB HDD	64 GB eMMC	32 GB MicroSD	64 GB eMMC
Operative System	Ubuntu 18.04.3 LTS (64 bit)	Ubuntu Linaro (32 bits)	Raspbian (32 bits)	Ubuntu Mate (64 bits)

**Table 3:** Navigation message generation setup. © [2020] IEEE. Reprinted, with permission, from [16].

	Parameter	Description	Sets of Values			
			S1 [17]	S2	S3	S4
OSNMA parameters	NS	Number of Satellites with different keys per MACK block	36			
	NB_KROOT	Number of 104-bit blocks of the DSM-KROOT	7		10	13
	NMACK	Number of MACK blocks within a subframe	2			
	HF	Hash Function used for the TESLA chain generation	SHA-256			
	MF	MAC Function used to authenticate the navigation data	HMAC-SHA-256			
	KS	Keys Size	96 bits			
	MS	MACs size	10 bits			
	MO	MACK Offset	0 (No offset)			
	ADKD	Authentication Data and Key Delay defining the pieces of information in the navigation message to be authenticated and the key to be used for it.	{0, 2, 3, 4, 11, 12}			
	NB_PKR	Number of 104-bit blocks of the DSM-PKR	13		14	16
	NPKT	New Public Key Type	ECDSA P224/SHA-224	ECDSA P256/SHA-256	ECDSA P384/SHA-384	ECDSA P521/SHA-512
	DSMs Sequence	Generation sequence of the DSMs	{DSM-KROOT, DSM-PKR, DSM-KROOT}			
	D_KROOT	Key root distance from the simulation start time	32 m 11 s			
General parameters	Number of Galileo Satellites	Number of generated Galileo Satellites	7			
	Galileo PRNs	Generated Galileo PRNs	{5, 6, 7, 14, 24, 25, 26}			
	NavMsg Length	Length of the generated nav. message	1 h			

**Table 4:** Software profiling analysis results obtained with the set of parameters S1 in Table 3. © [2020] IEEE. Extended, with permission, from [16].

	Platform 1 [16,17]				Platform 2 [16]			Platform 3			Platform 4		
	Call Rate (Hz)	avr (µs)	σ (µs)	ε (%)	avr (µs)	σ (µs)	ε (%)	avr (µs)	σ (µs)	ε (%)	avr (µs)	σ (µs)	ε (%)
TESLA key verification (one step)	13.60	0.61	0.02	2.87	2.77	0.50	18.16	2.66	0.09	3.44	2.70	0.07	2.44
MAC verification	2.62	7.85	0.21	2.66	68.36	2.44	3.57	45.69	6.19	13.54	47.34	0.91	1.91
MACSEQ verification	0.27	5.84	0.18	3.11	44.54	5.05	11.35	40.94	0.60	1.48	40.92	0.92	2.24
Digital Signature verification	0.03	134.82	1.51	1.12	2835.13	6.64	0.23	1857.40	11.63	0.62	1416.60	18.25	1.29
Public key verification	0.01	2.76	0.08	3.08	25.46	0.35	1.38	14.36	0.50	3.52	11.30	1.03	9.09

**Table 5:** Execution time degradation factors in terms of mean value avr and standard deviation σ on the three embedded boards, i.e., platforms 2, 3 and 4, with respect to the standard PC, i.e., platform 1, for all the OSNMA functions reported in Table 4.

	Platform 2		Platform 3		Platform 4	
	avr	σ	avr	σ	avr	σ
TESLA key verification (one step)	4.54	25.00	4.36	4.50	4.43	3.50
MAC verification	8.71	11.62	5.82	29.48	6.03	4.33
MACSEQ verification	7.63	28.10	7.01	3.33	7.01	5.11
Digital Signature verification	21.02	4.40	13.78	7.70	10.51	12.09
Public key verification	9.22	4.37	5.20	6.25	4.09	12.87

**Table 6:** Software profiling analysis results of the digital signature verification and its subroutines obtained on the three embedded platforms with the set of parameters S1 in Table 3. © [2020] IEEE. Extended, with permission, from [16].

		Call Rate (Hz)	Platform 2 [16]			Platform 3			Platform 4		
			avr (µs)	σ (µs)	ε (%)	avr (µs)	σ (µs)	ε (%)	avr (µs)	σ (µs)	ε (%)
Calling function	Digital Signature verification	0.03	2835.13	6.64	0.23	1857.40	11.63	2.70	1416.60	18.25	1.29
Subroutines	Asn1 encoding		1.24	0.06	4.81	1.41	0.09	6.49	0.85	0.13	15.36
	EVP Verify Init		4.05	0.19	4.70	3.97	0.23	5.84	6.74	1.28	19.05
	EVP Verify Update		0.86	0.03	3.58	1.19	0.08	7.17	0.67	0.12	17.99
	EVP Verify Final		2820.23	6.42	0.23	1841.07	11.43	0.62	1400.28	15.40	1.10

**Table 7:** Execution time profiling of the digital signature verification obtained on the three embedded platforms with the sets of values S1 to S4 in Table 3. © [2020] IEEE. Extended, with permission, from [16].

		Platform 2 [16]			Platform 3			Platform 4		
**Digital Signature verification**	**Sets of Values**	avr (µs)	σ (µs)	ε **(%)**	avr (µs)	σ (µs)	ε **(%)**	avr (µs)	σ (µs)	ε **(%)**
	S1	2835.13	6.64	0.23	1857.40	11.63	0.62	1416.60	18.25	1.29
	S2	932.90	4.34	0.46	706.54	5.75	0.81	457.51	13.62	2.98
	S3	10,189.00	16.97	0.17	5959.01	31.05	0.5	3997.05	31.35	0.78
	S4	22,982.28	16.28	0.07	14,074.56	61.98	0.44	9419.61	18.82	0.20

**Table 8:** Test setup.

GNSS Signal Generator Setup	
**GNSS Signals**	10 GPS L1 and 6 Galileo E1
**Galileo OSNMA setup**	set of values *S4* in Table 3
**GNSS Received Power**	−110 dBm for all GNSS signals
**Simulated User Dynamic**	Static Position
**FE Configuration**	
**FE**	SiGe v2 [28]
**Sampling frequency** $f_{s}$ **(MHz)**	16.367
**Intermediate frequency** $f_{IF}$ **(MHz)**	4.1304

**Table 9:** Call rate and average execution time of the main receiver processing steps, configured to elaborate 12 satellites, i.e., 6 GPS and 6 Galileo.

		Call Rate (Hz)	Average Execution Time (µs)			
			Platform 1	Platform 2	Platform 3	Platform 4
GPS	Coarse acquisition	1000	45	716	423	607
	Doppler Refinement		6	77	37	55
	Code Refinement		6	76	37	56
	Confirmation		6	77	37	54
	Tracking		8	125	74	96
Galileo	Coarse acquisition	250	90	1178	751	897
	Doppler Refinement		27	381	186	280
	Code Refinement		27	376	185	282
	Confirmation		27	378	185	279
	Tracking		36	567	340	432
	PVT computation	1	2081	30,529	16,907	16,222

**Table 10:** Execution time degradation factors on the three embedded boards, i.e., platforms 2, 3 and 4, with respect to the standard PC, i.e., platform 1, for all the main processing steps reported in Table 9.

		Platform 2	Platform 3	Platform 4
GPS	Coarse acquisition	15.91	9.4	13.49
	Doppler Refinement	12.83	6.17	9.17
	Code Refinement	12.67	6.17	9.33
	Confirmation	12.83	6.17	9
	Tracking	15.62	9.25	12
Galileo	Coarse acquisition	13.09	8.34	9.97
	Doppler Refinement	14.11	6.89	10.37
	Code Refinement	13.96	6.85	10.44
	Confirmation	14	6.85	10.33
	Tracking	15.75	9.44	12
	PVT computation	14.67	8.12	7.79

**Table 11:** CPU and RAM usage summary.

Platform	CPU Usage		RAM Usage
	Average (%)	Maximum (%)	Maximum (MiB)
1	39.7	44.0	24.3
2	266.9	297.0	148.0
3	173.7	204.3	151.0
4	244.0	276.4	27.5

## Data Availability

The data presented in this study are available in this published article.
